# Using the hierarchical ordinal regression model to analyse the intensity of urinary schistosomiasis infection in school children in Lusaka Province, Zambia

**DOI:** 10.1186/s40249-017-0262-x

**Published:** 2017-02-21

**Authors:** Christopher Simoonga, Lawrence N. Kazembe

**Affiliations:** 1 Ministry of Health, Ndeke House, Haile Selassie Avenue, P.O. Box 30205, Lusaka, Zambia; 2 Mathematical Sciences Department, Chancellor College, University of Malawi, P.O. Box 280, Zomba, Malawi; 3 Statistics and Population Studies Department, University of Namibia, Private Bag 13301, Windhoek, Namibia

**Keywords:** Urinary schistosomiasis, Ordinal logistic regression, Intensity of infection, Zambia, Bayesian analysis

## Abstract

**Background:**

Urinary schistosomiasis has been a major public health problem in Zambia for many years. However, the disease profile may vary in different locale due to the changing ecosystem that contributes to the risk of acquiring the disease. The objective of this study was to quantify risk factors associated with the intensity of urinary schistosomiasis infection in school children in Lusaka Province, Zambia, in order to better understand local transmission.

**Methods:**

Data were obtained from 1 912 school children, in 20 communities, in the districts of Luangwa and Kafue in Lusaka Province. Both individual- and community-level covariates were incorporated into an ordinal logistic regression model to predict the probability of an infection being a certain intensity in a three-category outcome response: 0 = no infection, 1 = light infection, and 2 = moderate/heavy infection. Random effects were introduced to capture unobserved heterogeneity.

**Results:**

Overall, the risk of urinary schistosomiasis was strongly associated with age, altitude at which the child lived, and sex. Weak associations were observed with the normalized difference vegetation index, maximum temperature, and snail abundance. Detailed analysis indicated that the association between infection intensities and age and altitude were category-specific. Particularly, infection intensity was lower in children aged between 5 and 9 years compared to those aged 10 to 15 years (*OR* = 0.72, 95% *CI* = 0.51–0.99). However, the age-specific risk changed at different levels of infection, such that when comparing children with light infection to those who were not infected, age was associated with a lower odds (category 1 vs category 0: *OR* = 0.71, 95% *CI*: 0.50–0.99), yet such a relation was not significant when considering children who were moderately or heavily infected compared to those with a light or no infection (category 2 vs category 0: *OR* = 0.96, 95% *CI*: 0.45–1.64). Overall, we observed that children living in the valley were less likely to acquire urinary schistosomiasis compared to those living in plateau areas (*OR* = 0.48, 95% *CI*: 0.16–0.71). However, category-specific effects showed no significant association in category 1 (light infection), whereas in category 2 (moderate/high infection), the risk was still significantly lower for those living in the valley compared to those living in plateau areas (*OR* = 0.18, 95% *CI*: 0.04–0.75).

**Conclusions:**

This study demonstrates the importance of understanding the dynamics and heterogeneity of infection in control efforts, and further suggests that apart from the well-researched factors of *Schistosoma* intensity, various other factors influence transmission. Control programmes need to take into consideration the varying infection intensities of the disease so that effective interventions can be designed.

**Electronic supplementary material:**

The online version of this article (doi:10.1186/s40249-017-0262-x) contains supplementary material, which is available to authorized users.

## Multilingual abstracts

Please see Additional file 1 for translations of the abstract into five official working languages of the United Nations.

## Background

Urinary schistosomiasis caused by the trematode *Schistosoma haematobium* has been a major public health problem in Zambia for many years [1, 2]. The infection affects people of all ages, however, children bear a huge burden [3]. Indeed, efforts to control the disease have focused on mass treatment of school-aged children, with praziquantel (PZQ) advocated for schools where the prevalence of the disease is 50% or higher. This treatment may be repeated annually to ensure that levels of infection are kept below the levels associated with severe morbidity [4]. However, in communities where reinfection rates are very high, chemotherapy alone may not suppress morbidity and needs to be combined with other interventions such as health education, improvement in the water supply and sanitation, and control of intermediate host snails where applicable [5, 6].

Although prevalence of infection is often used to guide control programmes, it is argued that the intensity of infection is more relevant to understanding transmission dynamics [7–9], since it is generally affected by environmental factors and show great seasonal fluctuations. Moreover, control programmes can have demonstrated impact on intensity of infection than prevalence of infection [10]. Therefore, the intensity of infection can be used to assess the effectiveness of interventions and is essential in deciding whether annual provision of mass treatments with PZQ should be continued [11, 12].

In this study, we developed a statistical model in order to estimate the intensity of infection with urinary schistosomiasis using data collected from two administrative districts in Lusaka Province, Zambia. The main aim of the study was to understand the epidemiology of urinary schistosomiasis in order to support the School Health and Nutrition Programme and the National Bilharzia Control Programme in the country. This includes identification of zones where the risk is high for prioritizing interventions, and designing health education campaigns that are to provide information on the disease, and possible adoption of preventive measures, as well as necessitate behavioural change.

We adapted the approach by Tarafder et al. [9] of using the number of eggs per milliliter (epm) of urine, divided into three category outcomes, to investigate risk factors associated with each level of intensity. In effect, a cumulative ordinal regression model was used to estimate the effects of individual-level variables, such as age and sex, and location-level environmental variables. Because of the small-scale focality of the disease, random effects were introduced to capture any heterogeneity that may exist in the outcome. This is the first time a study like this has been conducted in Zambia and results might lead to an improvement in the understanding of the transmission dynamics of *S. haematobium* in this part of the country.

## Methods

### Study area and design

**Fig. 1:**
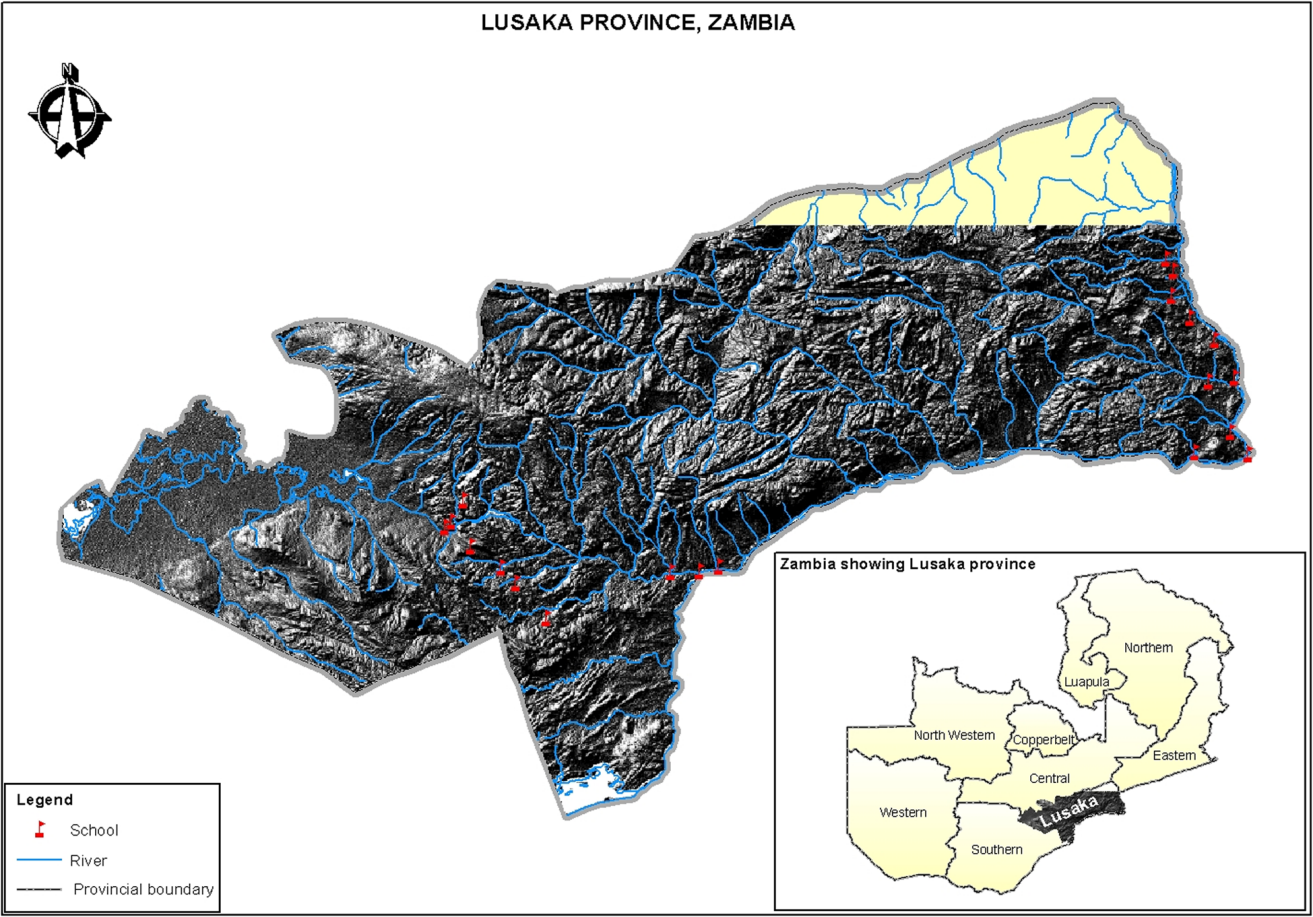
The study areas and its position in Zambia (shaded region in the insert)

The data were collected as part of a cross-sectional study carried out in two administrative districts, Kafue and Luangwa, in Lusaka Province, Zambia (see Fig. 1), after obtaining ethical clearance from the University of Zambia Ethics Committee. The two districts were selected on the basis of their ecological representativeness of the country [13, 14]. In each of these districts, 10 primary schools were selected. Approximately 100 school children, aged 6 to 15 years, were recruited from each school in both districts. The altitude and geographical location (longitude, latitude) of the surveyed schools were obtained from the archives of the Survey Department (2003). Further details of the study design are given elsewhere [15].

### Field data collection

Data on *S. haematobium* intensity were obtained using the quantitative filtration technique [16]. About mid-morning, urine samples were collected from the pupils, and duplicate filters were prepared and examined microscopically. Two laboratory technicians were trained to prepare and read the specimen filters. Both technicians read each specimen independently. This was useful for increasing the sensitivity of the technique, particularly where egg intensity was low [17]. All pupils found infected were treated with PZQ (40 mg/kg body weight). Individual datasheets were used to collect ancillary information on each child. This information included demographic, water contact and personal hygiene variables.

In addition, data on intermediate host snails were obtained through field collections and laboratory-based species identification. The sampling of potential schistosomiasis transmission sites was done based on the proximity of the water body to the respective primary school, i.e. the nearest likely infection source. These water points were also qualified by relevant local people as the most frequented water contact points for both domestic use and/or livestock.

The identified sites were geo-referenced using a handheld global positioning system (GPS) [18]. The prevalence and abundance of intermediate host snails were assessed during malacological surveys conducted before and after the rainy season. Two field operators were allocated a duration of 15 min each for scooping using standardized snail scoops during the snail surveys. Where scoops were not useable, for instance, in muddy semi-dry habitats, the operators handpicked snails for 15 min each.

Collected snails were placed individually in vials containing 10 ml of water and exposed to light for two hours in order to induce cercarial shedding. Due to a lack of facilities for species identification of cercariae, this study used morphology and rhythmic vibrations to differentiate between shaded human and mammalian cercariae, as previously described by Jordan and Webbe [7] and Davis et al. [19]. Snails that were shedding were placed in separate vials for species identification using field guides [20, 21].

### Climatic data

Climatic data, of 1-km image files, were downloaded from the website [22]. These images were captured by the Advanced Very High Resolution Radiometer onboard the National Oceanic and Atmospheric Administration polar-orbiting meteorological satellites [23]. The data were then calibrated into normalized difference vegetation index (NDVI) and midday earth surface temperature (T_*max*_) values using the ERDAS Imagine 8.5 software [24] for each 10-day interval between April 1992 and September 1993, and between February 1995 and January 1996.

### Statistical analysis

#### Descriptive analysis

A three-category response variable was analysed using a cumulative ordinal regression model. The response was categorized as follows: no infection (0 epm of urine), light infection (1–100 epm of urine), and moderate/heavy infection (>100 epm of urine). This categorization was based on the World Health Organization (WHO) standard [9, 25], however, the categories of moderate and heavy infection were combined because of the small number of children in those groups. The outcome was further stratified by altitude where the child lived (elevation of 601–1 150 m for plateau and < 601 m for valley); sex (male, female); and age (6–9 years and 10–15 years); and assessed for any significant variations. The chi-square test was used to determine whether there were any associations between the intensity of infection and altitude, sex, and age. The analyses were carried out using the R software [26].

#### Hierarchical modeling

Three cumulative ordinal regression models were developed to determine the relationship between the intensity outcome, as defined above, and risk factors, i.e. environmental/ecological (altitude, NDVI, T_*max*_); malacological (host snail abundance); and individual-level demographic covariates (sex, age).

The first model fitted was a cumulative ordinal regression model without adjusting for clustering, i.e., we assumed homogeneity in the infection rates across communities. The second model was an extension of the first and included random effects to capture unstructured heterogeneity. To model the unstructured heterogeneity, we assumed an exchangeable Gaussian processes. The third model was a cumulative model with category-specific fixed effects. Here, age and altitude were estimated as fixed effects corresponding to each of the comparative categories. A further modification to the third model was made by fitting smooth (non-linear) effects of age and altitude, which were estimated non-parametrically [27].

The model building strategy considered the same fixed effects covariates with and without random effects. The three models were then compared using the Akaike information criteria (AIC), with small values of AIC implying a better fitting model. All cumulative logit models were estimated in BayesX using the restricted maximum likelihood regression procedure [27]. Model validation used receiver operating characteristic (ROC) curves analysis, a method recently used to validate regression models [14], in which the proportion of true positives (sensitivity) is plotted against the proportion of false negatives (1-specificity) across a range of threshold values. One performance measure used in the ROC analysis is the area under the curve (AUC) of the ROC plot. A purely random model would be expected to be correct half the time (AUC = 0.5), whereas a perfect model would be correct all the time (AUC = 1.0). We validated the ordinary model (model 1) against the random effects models (models 2 and 3).

## Results

Table 1 summarizes the characteristics of the study population. A total of 2 040 school children aged 6 to 15 years were enrolled into the study from 20 selected primary schools in the two districts, Kafue and Luangwa, of which 1 912 (94%) provided urine samples for parasitological examination.

**Table 1:** Characteristics of 2 040 children, and intensity of infection with *S. haematobium* in 1 912 children from 20 schools in Lusaka Province, Zambia, 2004

Variable	Mean (St. Dev)	Number (%)
Intensity of infection		
No infection (0 eggs/ml: epm)		1 726 (84.6)
Light infection (1 – 100 epm)		145 (7.1)
Moderate/heavy infection (>100 epm)		40 (2.1)
Age		9.98 (2.2)
6–9 years		1 130 (55.9)
10–15 years		900 (44.1)
Sex		
Female		1 027 (50.4)
Male		1 000 (49.6)
Altitude		
Plateau		723 (35.5)
Valley		1 316 (64.5)
NDVI	138.6 (4.9)	
T_*max*_	19.6 (3.1)	
Snail abundance (*B. globosus*)	25.3 (37.1)	

**Fig. 2:**
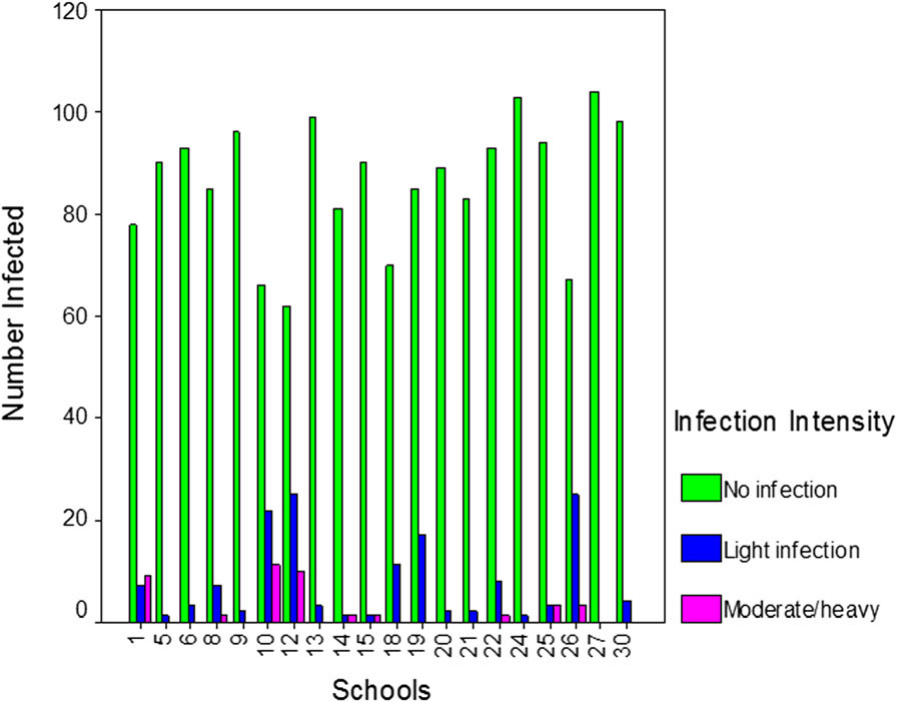
Number of children infected in each of the three intensity of infection categories, by school/community

The overall prevalence rate for the two districts was 9.6% (range: 0–36.1%), with the prevalence in Kafue slightly higher than that in Luangwa (10.9% vs. 8.4%), although this was not significant. The intensity of infection had a mean of 31.4 eggs/10 ml of urine (range: 0–120 eggs/10 ml), and a significant difference in the mean intensity of infection was observed, with 40.2 eggs/10 ml (range: 3–53.1 eggs/10 ml) observed in Kafue and 22.6 eggs/10 ml (range: 0–116.0 eggs/10 ml) in Luangwa. Significant differences in infection intensities were also noted among communities, ranging from 0 to 100% (see Fig. 2).

**Fig. 3:**
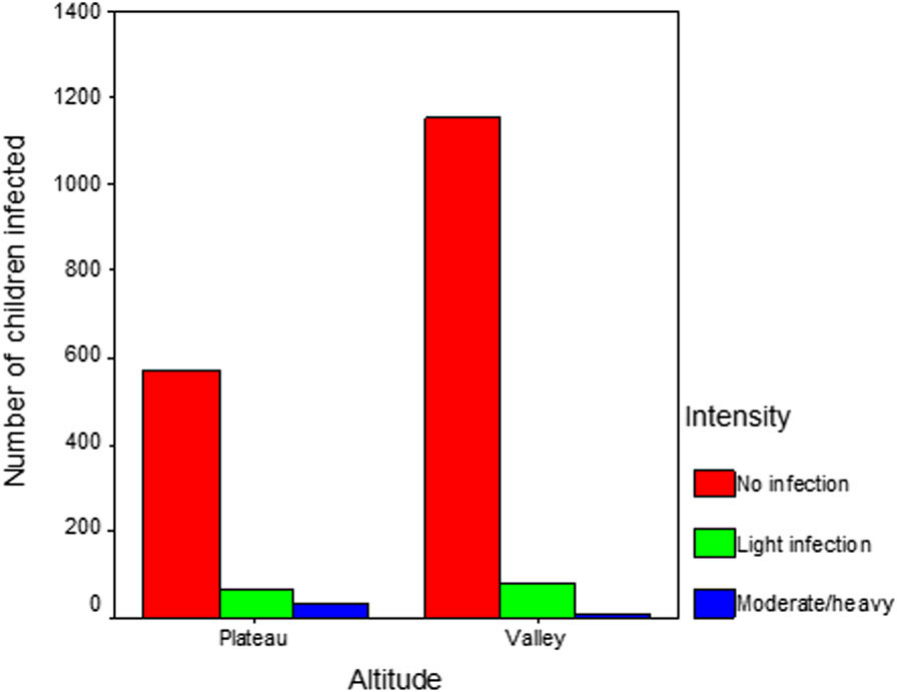
Number of children infected in each of the three intensity of infection categories, by altitude

Table 2 shows the association between intensity of infection with the children’s ages, sex, and altitude of village where the child lived. No significant differences in intensity of infection were observed between the two age groups (*χ* ^2^ = 4.1, *P* = 0.13) and sex (*χ* ^2^ = 2.5, *P* = 0.29). However, significant differences in intensity of infection were observed with altitude sex (*χ* ^2^ = 42.7, *P* = 0.001). Figure 3 also shows variability in the number of children infected at each intensity level with respect to altitude.

**Table 2:** Associations between intensity of infection and sex, age, and altitude, obtained using the chi-square test

	Intensity of infection (N and %)			
	No infection	Light infection	Moderate/heavy infection sex *χ* ^2^	(*P*-value)
Variables				
Age				
6–9 years	953 (89.1)	93 (8.7)	23 (2.2)	4.1 (0.13)
10–15 years	765 (91.7)	52 (6.2)	17 (2.0)	
Sex				
Female	871 (91.3)	66 (6.9)	17 (1.8)	2.5 (0.29)
Male	843 (89.2)	79 (8.4)	23 (2.4)	
Altitude				
Plateau	570 (85.3)	67 (10.0)	31 (4.6)	42.7 (0.001)
Valley	1 156 (93.0)	78 (6.3)	9 (0.7)

Table 3 gives the AIC for the three estimated models. Model 3 had the lowest log-likelihood (LL) and AIC, and therefore the best fitting (LL = 1 147.77 and AIC = 1 198.28 in model 3 vs. LL = 1 366.64 and AIC = 1 382.64 in Model 1). Moreover, by considering the difference in AIC between Model 2 and 3 (∆*AIC*=6.88) implies that assuming a category-specific model further improved the model fit to the data.

**Table 3:** Estimated *OR*s of factors associated with the prevalence of light and at least moderate intensities of infection obtained from the cumulative logit models

	Model 1	Model 2	Model 3
Variable	OR (95% CI)	OR (95% CI)	OR (95% CI)
Sex			
Male	1.19 (0.88, 1.63)	1.52 (1.09, 2.13)	1.53 (1.09, 2.10)
Female	1.00	1.00	
NDVI	1.04 (1.00, 1.07)	1.01 (0.96, 1.05)	1.01 (0.96, 1.05)
T_*max*_	0.99 (0.93, 1.04)	1.00 (0.94, 1.07)	1.00 (0.94, 1.07)
Snail abundance			
	1.00 (1.00, 1.05)	1.01 (1.00, 1.01)	1.01 (1.00, 1.01)
Age			
5–9 years	0.69 (0.51, 0.96)	0.72 (0.51, 0.99)	Cat1:0.71 (0.50, 0.99)
			Cat2:0.96 (0.45, 1.64)
10–20 years	1.00	1.00	1.00
Altitude			
Valley	0.36 (0.25, 0.51)	0.48 (0.16, 0.71)	Cat1:0.49 (0.14, 1.75)
			Cat2:0.18 (0.04, 0.75)
Plateau	1.00	1.00	1.00
Model selection			
LL	1 366.64	1 163.35	1 147.77
AIC	1 382.64	1 205.16	1 198.28

Cat1: Category 1 (light infection); Cat2: Category 2- moderate/high infection; LL: likelihood; AIC: Akaike Information Criterion

Table 3 also shows the odds ratios (ORs) estimated using Model 3. However, the results of Model 2 are also presented as these give overall effects and for comparison with those obtained in Model 3. The OR compares individuals at least lightly infected (>0 epm) to individuals not infected (0 epm), and individuals moderately or heavily infected (>100 epm) to individuals lightly infected or not infected (0–100 epm). Overall, the risk of urinary schistosomiasis was found to be lower in children aged between 5 to 9 years compared to those aged 10 to 20 years (*OR* = 0.72, 95% confidence interval (*CI*) = 0.51–0.99). The risk, however, changed when comparing the effect of age on children with light infections to those not infected (category 1), and between those moderately or heavily infected to those with light or no infection (category 2). In category 1, the effect of age was significantly lower (OR = 0.71, 95% *CI*: 0.50–0.99), whereas in category 2, the effect of age was lower but not significant (OR = 0.96, 95% *CI*: 0.45–1.64).

Children living in the valley were less likely to acquire urinary schistosomiasis compared to those living in plateau areas (*OR* = 0.48, 95% *CI*: 0.16–0.71). Category-specific effects showed that in category 1, the risk was not significant, although lower for children living in valleys compared to those living in plateau areas (*OR* = 0.49, 95% *CI*: 0.14–1.75). In category 2, the risk was still significantly lower for those living in the valley compared to those living in plateau areas (*OR* = 0.18, 95% *CI*: 0.04–0.75).

Increased risk of urinary schistosomiasis was also observed in male children (*OR* = 1.53, 95% *CI*: 1.09–2.10). We also observed a positive relationship between snail abundance and risk of infection, significant at 5% (*OR* = 1.01, 95% *CI*: 1.00–1.01). However, marginal positive associations were observed between urinary schistosomiasis and NDVI (the mean Dec – Nov biannual composites of NDVI) (*OR* = 1.01, 95% *CI*: 0.96–1.05), as well as with T_max_ (*OR* = 1.00, 95% *CI*: 0.94–1.07).

**Fig. 4:**
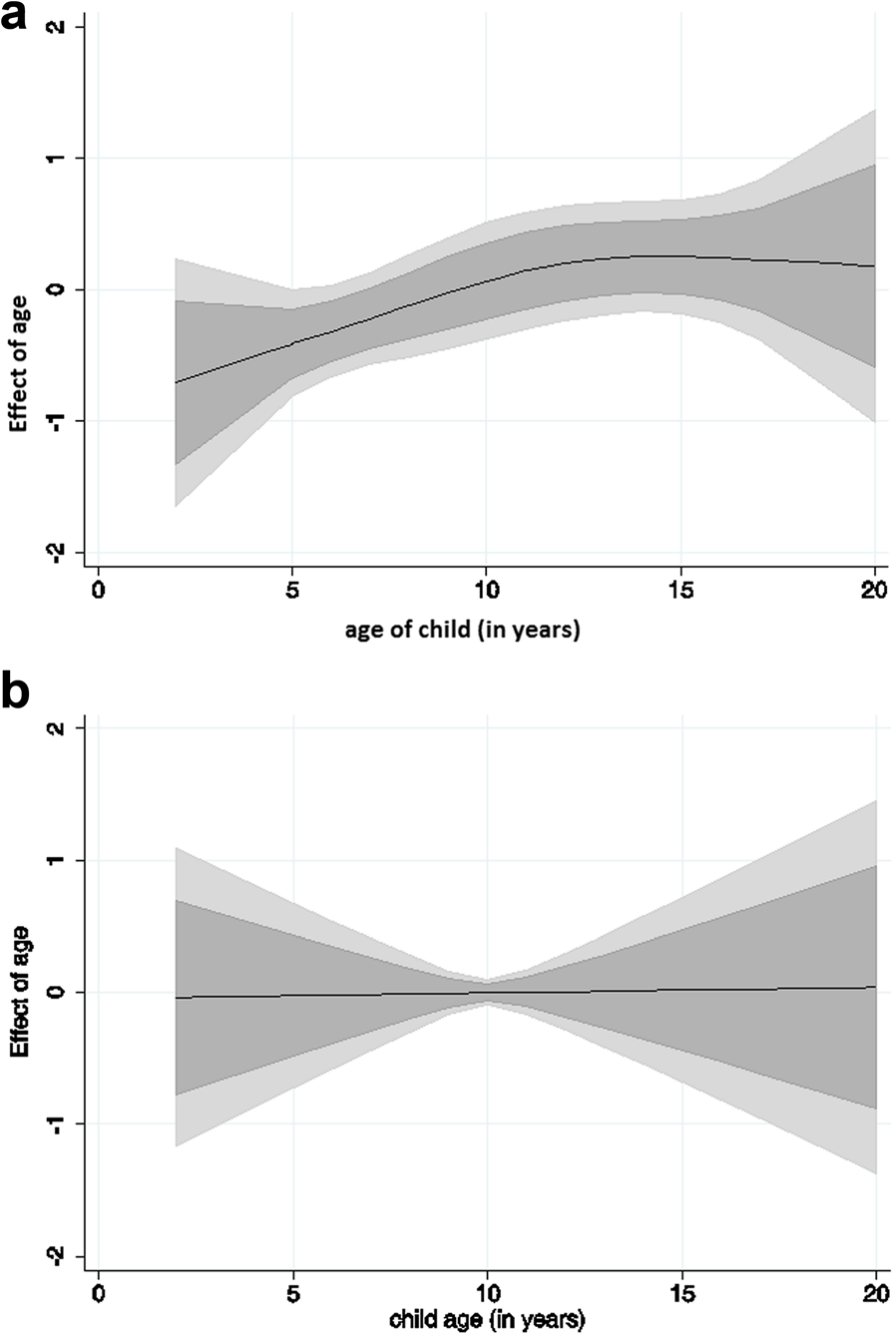
Smooth effects of age (middle line) on the intensity of infection (a) for light infection (category 1) shown in plot (**a**); and (**b**) moderate/high infection (category 2) shown in panel (b). The outer two lines in both plots represent the corresponding confidence bands at 80% (inner lines from the middle line) and 95% (outer lines)

Figure 4 shows the nonlinear effects of age for categories 1 and 2. In the first category, the effect of age showed some form of non-linearity (middle line). Particularly, the risk increased with rising age up to 12 years, and then dropped slightly and remained constant up until 20 years. In the second plot, we show the effects of age in category 2. The age effect in the plot was linear, and the risk was comparatively similar for children of all ages. This is not surprising since the results for category 2 in Table 3 confirm a non-significant association.

**Fig. 5:**
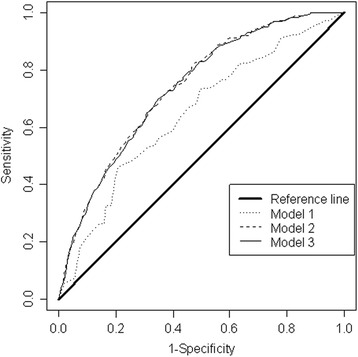
ROC analysis of the ordinary and random effects of urinary schistosomiasis prevalence. The solid black line is the reference line that represents equal trade-off of sensitivity and specificity of the model

Figure 5 shows the ROC analysis results for the three models. The solid black reference line represents equal trade-offs between the sensitivity and specificity of the predictivity of the model. The area under the ROC curve of the reference line is equal to 0.500. The AUC for the ordinary logistic regression model (model 1) is 0.61, while for the two random effects models it is 0.738 (model 2) and 0.736 (model 3). The overall predictive performance of the random effects models was higher than the ordinary model. The AUC of 0.738 in model 2 implies that the prevalence of urinary schistosomiasis infection can be as high as 73.8%.

## Discussion

It is crucial to have insights into the epidemiology of schistosomiasis in order to achieve its successful control using mass chemotherapy with PZQ. For example, a PZQ dose of 40 mg/kg body weight has been reported to have cure rates ranging from 70 to 99% for *S. haematobium* infections [28, 29]. However, low cure rates are possible, and these have been attributed to high initial worm loads and intense transmission in an area [30]. In places where the disease is endemic, for instance in Zambia, it is imperative to study the epidemiological factors related to the intensity of infection [11].

This study used an ordinal regression model to quantify factors associated with the intensity of *S. haematobium* infection among school children in Lusaka Province. The intensity of infection was derived by categorizing egg counts into three groups, based on egg counts in the urine samples, namely of no infection, light infection, and moderate/high infection. An alternative approach to modeling intensity is using the actual egg counts and applying a negative binomial model [8].

The proportion of children with moderate to high infection was very small (2.1%) compared to those with no infection (84.6%). Nevertheless, the dynamics of infection were clearly different for different ages, and at diverse locations and altitudes. Children in the younger age category (5–9 years) were found to have a higher intensity of infection than those in the older age range (10–15 years). This could be attributed to the higher infection risk behaviors of younger children compared to older ones. Our findings are consistent with studies conducted by Tingley et al. [31] and Estard et al. [32], who found higher infection intensities in younger children than in older children. Schools located in plateau areas were found to have higher infection rates, which is contrary to findings in many reports, since the climatic and environmental conditions for schistosomes and the different intermediate host snails are not favorable for transmission at high altitudes [33, 34]. However, the findings are not totally surprising and do agree with recent studies from Uganda [35]. *Schistosoma* transmission, it is argued, is due to the availability of suitable host snails, for example, *Biomphalaria pfeifferi,* which prefer temporary water bodies. Thus the continuing schistosomiasis transmission at high altitudes is sustained because of such environments (temporary water bodies) which supports snail’s presence and abundance [35]. Indeed, the findings reported here also identified snail abundance as a factor associated with the varying intensities of infection. Indeed snails are easily affected by environmental factors such as vegetation abundance, as measured by the NDVI, and temperature regimes. The role of environmental factors in defining the intensity of infection are well recognized, and a combination of integrated environmental control and chemotherapy is needed to achieve sustainable transmission control [36–38], and thus are crucial for guiding schistosomiasis control.

The improvement in the model after accounting for random effects (model 3) confirmed the presence of spatial clustering or small-scale heterogeneity of schistosomiasis infection [39]. Our analysis could benefit from using Bayesian methods to account for such spatial dependence, however, the sampling design was not optimized for spatial analysis [40, 41].

The significance of random effects further suggests that apart from well-researched factors, for example, the ones mentioned above, various unobserved agents influence the complexity of transmission accounting for the differences in schistosomiasis infection. An immediate example worth considering is water contact behavior, which is critical for transmission among school-aged children [42]. Heterogeneity may similarly be regulated by varying socio-cultural factors, which are also important to explore in order to properly target control regimes in a community; however, such studies are rare and usually comprise just a small component of large baseline studies conducted by control programmes [5, 39]. When available, such variables should be included in the model.

Although carried out using an exploratory approach, the category-specific effects of age and altitude on the intensity of infection do suggest an interesting epidemiological finding, but with broader implications for disease control. The immediate implication is that the epidemiology of schistosomiasis is complex. Evidently, there is a dynamic effect of risk factors with varying intensity of infection. In other words, the risk may vary at different levels of infection (model 3), but may also change at different levels of the risk factor, especially for continuous covariates such as age (Fig. 4a).

## Conclusions

Control programmes need to take into consideration the varying intensity of infection. This will help to design and deploy cost-effective interventions. Higher infection intensity might require more frequent treatment regimes. It may also imply combining interventions for morbidity control using chemotherapy and controlling of intermediate host snails, in order to ensure sustainability. Use of models to find hotspots of urinary schistosomiasis of different intensities is necessary. This may assist in informing surveillance and response systems for the elimination and control of re-emerging tropical diseases such as schistosomiasis [43–46].

## Additional file

Additional file 1: Multilingual abstract in the five official working languages of the United Nations. (PDF 656 kb)

## Abbreviations

AIC: Akaike information criteria
AUC: Area under the curve
CI: Confidence interval
epm: Eggs per milliliter
LL: Log-likelihood
NDVI: Normalized difference vegetation index
OR: Odds ratio
PZQ: Praziquantel
ROC: Receiver operating characteristic
