# Supplementary material for: Using the hierarchical ordinal regression model to analyse the intensity of urinary schistosomiasis infection in school children in Lusaka Province, Zambia
Source: Infect Dis Poverty. 2017 Feb 21;6:43. doi: 10.1186/s40249-017-0262-x (PMC5319044; doi:10.1186/s40249-017-0262-x)
Supplement: Additional file 1: — Multilingual abstract in the five official working languages of the United Nations. (PDF 656 kb) [file 40249_2017_262_MOESM1_ESM.pdf]

Translation of the abstract into the five official working languages of the United Nations

استخدام نموذج الانحدار الترتيبي الهرمي لتحليل شدة الإصابة بالبلهارسيا البولية لدى أطفال المدارس في مقاطعة لوساكا، زامبيا

كريستوفر سيمونجا، لورانس ن. كازيمبي

ملخص

خلفية: شكلت البلهارسيا البولية مشكلة صحية عامة رئيسية في زامبيا لسنوات عديدة. ومع ذلك، قد يختلف شكل المرض في الأماكن مختلفة بسبب تغير النظام البيئي الذي يساهم في مخاطر اكتساب المرض. وكان الهدف من هذه الدراسة هو تحديد عوامل الخطر المرتبطة بشدة الإصابة بالبلهارسيا البولية لدى أطفال المدارس في مقاطعة لوساكا، زامبيا، من أجل فهم أفضل انتقال المرض في البيئة المحلية.

الأساليب: تم الحصول على بيانات 1912 من أطفال المدارس، في 20 مجتمعا محليا، في مناطق وانجوا وكافو في مقاطعة لوساكا. أدرجت المتغيرات المصاحبة على المستوى الفردي وعلى مستوى المجتمع المحلي في نموذج انحدار لوجستي ترتيبي للتنبؤ باحتمال وجود عدوى عند كثافة معينة وفق ثلاث فئات: 0 = لا عدوى، 1 = عدوى بسيطة، 2 = عدوى معتدلة/شديدة. تم إدخال التأثيرات العشوائية لالتقاط التغاير غير الملحوظ.

النتائج: عموما، ارتبط خطر الإصابة بالبلهارسيا البولية بقوة مع العمر، ارتفاع مكان المعيشة الطفل، الجنس. وقد لوحظت ارتباطات ضعيفة مع مؤشر تطبيع الفرق للغطاء النباتي، درجة الحرارة القصوى، وانتشار الحلزون. وأشار تحليل مفصل إلى أن العلاقة بين شدة الإصابة والعمر والارتفاع كانت محددة الفئة. بشكل خاص، كانت شدة الإصابة أقل في الأطفال الذين تتراوح أعمارهم بين خمس وتسع سنوات مقارنة عن أولئك الذين تتراوح أعمارهم بين 10 إلى 15 سنة (OR = 0.72، 95٪ CI: 0.99-0.51). ومع ذلك، تغير الخطر المرتبط بالسن عند مستويات مختلفة من العدوى، بحيث عند المقارنة بين الأطفال الذين يعانون من إصابة بسيطة مع أولئك الذين لم يصابوا بالمرض، ارتبط عمر مع انخفاض احتمالات (الفئة 1 مقابل الفئة 0: OR =0.71، 95٪CI : 0.99-0.50)، ولكن كان مثل هذه العلاقة لم تكن ذات أهمية كبرى عند مراجعة الأطفال معتدلي الإصابة أو المصابين بشدة مقارنة مع المصابين بإصابة بسيطة أو غير المصابين من الأساس (الفئة 2 مقابل الفئة 0: OR = 0.96، 95٪ CI:1.64-0.45). عموما، لاحظنا أن الأطفال الذين يعيشون في الوادي كانوا أقل احتمالا بالإصابة بالبلهارسيا البولية مقارنة مع أولئك الذين يعيشون في مناطق الهضبة (OR = 0.48، 95٪ CI:0.71-0.16). ومع ذلك، لم تظهر الآثار المرتبطة بفئة محددة أي دلالة إحصائية في الفئة 1 (إصابة بسيطة)، في حين أن الفئة 2 (إصابة معتدلة/شديدة)، كان الخطر لا يزال أقل بكثير عن أولئك الذين يعيشون في الوادي مقارنة مع أولئك الذين يعيشون في مناطق الهضبة (OR = 0.18، 95٪ CI: 0.75-0.04).

الخلاصة: توضح هذه الدراسة أهمية فهم ديناميات وعدم التجانس الإصابة في جهود السيطرة على المرض، كما تشير إلى أنه وبصرف النظر عن العوامل المدروسة جيدا لشدة الإصابة بالبلهارسيا، هناك عوامل أخرى مختلفة تؤثر على انتقال المرض. تحتاج برامج المكافحة إلى أن تأخذ في الاعتبار تفاوت شدة الإصابة من هذا المرض بحيث يمكن إحداث أساليب تدخل فعالة للسيطرة على المرض.

Translated from English version into Arabic by Mahmoud Sami, through

TRANSLATORS WITHOUT BORDERS

分层次序回归模型分析赞比亚卢萨卡省学龄儿童尿路血吸虫病感染度

Christopher Simoonga, Lawrence N. Kazembe

摘要

引言：多年来，尿路血吸虫病一直是赞比亚的一个主要公共卫生问题。然而，由于不断变化的生态系统有助于增加患病风险，因此，在不同地区该病概况可能会有变化。本研究的目的是量化与赞比亚卢萨卡省儿童尿路血吸虫病感染强度相关的危险因素，以便更好地了解当地传播情况。

方法：从卢萨卡省 Luangwa 和 Kafue 地区的 20 个社区的 1 912 名学童中获取数据。将个体水平和群体水平的协变量结合到分层次序回归模型中，以预测在三类结果中感染强度的概率：0 =感染，1 =轻度感染，2 =中度/重感染。运用随机效应来获取研究中未观察到的异质性。

结果：总的来说，尿路血吸虫病的患病风险与年龄，儿童居住的海拔和性别密切相关。同时也观察到该疾病与归一化的植被指数，最高温度和钉螺丰度关联较弱。详细数据分析表明，感染强度与年龄和海拔之间的关联具有类别特异性。特别地，与 10 至 15 岁的儿童相比，5 至 9 岁的儿童的感染强度较低（*OR*＝0.72,95％*CI*＝0.51-0.99）。然而，年龄特异性风险在不同的感染水平上有所不同，比如当轻度感染的儿童与未感染的儿童比较时，年龄与该疾病患病风险有较低的相关性（1 类：0 类为 OR＝0.71,95％CI ：0.50-0.99），然而中度或重度感染的儿童与轻或无感染者相比，年龄与该疾病的相关性不显著，（第 2 类：第 0 类为 *OR*＝0.96,95％*CI*：0.45-1.64）。总体而言，与生活在高原地区的儿童相比，生活在山谷中的儿童患上尿路血吸虫病可能性小（*OR*＝0.48,95％*CI*：0.16-0.71）。然而，类别特异性效应表明，在类别 1（轻度感染）中，生活在山谷中的儿童与居住在高原地区的儿童相比，居住的海拔与该疾病患病风险无显著相关性；而在类别 2（中度/高度感染）中，与居住在高原地区的儿童相比，生活在山谷中的儿童患病风险较低降低（*OR* = 0.18,95％*CI*：0.04-0.75）。

结论：这项研究表明了解防控效果中感染动力学和异质性的重要性，并进一步表明，除了血吸虫感染强度的这个充分研究的因素意外，各种其他因素也会影响传播。控制疾病进程需要考虑到疾病的不同感染强度，以便制定出有效的干预措施。

Translated from English version into Chinese by Lei Sun, edited by Pin Yang

CDC CHINESE CENTER FOR DISEASE CONTROL AND PREVENTION
NATIONAL INSTITUTE OF PARASITIC DISEASES

Utilisation du modèle de régression ordinale hiérarchique pour analyser l'intensité de la schistosomiase urinaire chez les écoliers de la Province de Lusaka, en Zambie

Christopher Simoonga, Lawrence N. Kazembe

RESUME

Contexte: la schistosomiase urinaire est, depuis des années, un problème majeur de santé publique. Le profil de cette infection peut cependant varier d'un lieu à l'autre en raison des modifications de l'écosystème, qui contribuent au risque de contracter la maladie. Le but de cette étude est de quantifier les facteurs de risque associés à l'intensité de la schistosomiase urinaire chez les écoliers de la Province de Lusaka, en Zambie, afin de mieux comprendre la transmission locale.

Méthodes: Des données ont été recueillies sur 1912 écoliers de 20 communautés des districts de Luangwa et Kafue, dans la Province de Lusaka. Les covariables individuelles et communautaires ont été incluses dans un modèle de régression logistique ordinale pour estimer la probabilité d'une

infection se trouvant à une certaine intensité dans une réponse de résultats de trois catégories : 0=pas d'infection, 1=légère infection, and 2=infection modérée à sévère. Des effets aléatoires ont été inclus pour saisir l'hétérogénéité non-détectés.

Résultats: Globalement, le risque de schistosomiase urinaire a été fortement associé à l'âge, à l'altitude à laquelle l'enfant vit, et au sexe. De faibles associations ont été observées avec l'indice normalisé de différence de végétation, de température maximum, et de la profusion de limaçons. Une analyse détaillée a montré que l'association entre les intensités des infections, et l'âge et l'altitude, étaient d'une catégorie spécifique. En particulier, l'intensité de l'infection était moins élevée chez les enfants âgés de cinq à neuf ans que chez ceux âgés de 10 à 15 ans (*OR* = 0,72, 95%*CI* = 0,51–0,99). Cependant, le risque spécifique à l'âge se modifiait à différents niveaux d'infection, de telle façon qu'en comparant des enfants présentant une infection légère à ceux qui n'étaient pas infectés, l'âge était associé à des cotes plus basses (categorie 1 vs categorie 0: *OR*=0,71, 95%*CI*: 0,50–0,99), et pourtant un tel rapport n'était pas significatif lorsque nous considérons les enfants modérément ou sévèrement infectés, comparés à ceux ayant une infection légère ou pas d'infection (catégorie 2 vs catégorie 0: *OR*=0,96, 95%*CI*: 0,45–1,64). Globalement, nous avons observé que les enfants qui vivent dans la vallée sont moins susceptibles de contracter la schistosomiase urinaire que ceux qui vivent dans les régions du plateau (*OR*=0,48, 95%*CI*: 0,16–0,71). Cependant, les effets spécifiques à la catégorie n'ont montré aucune association significative dans la catégorie 1 (infection légère), alors que dans la catégorie 2 (infection modérée/élevée), le risque était encore considérablement moins élevé pour ceux vivant dans la vallée que pour ceux vivant dans les régions du plateau (*OR*=0,18, 95%*CI*: 0,04–0,75).

Conclusion: Cette étude démontre l'importance de comprendre la dynamique et l'hétérogénéité de l'infection dans les efforts de contrôle, et suggère en outre qu'à part les facteurs bien documentés de l'intensité de la *Schistosoma,* divers autres facteurs influencent la transmission. Les programmes de contrôle doivent tenir compte de l'intensité variable de l'infection de la maladie afin de concevoir des interventions efficaces.

Translated from English version into French by Ode Laforge, through

TRANSLATORS
WITHOUT BORDERS

Используя иерархическую модель регрессии Порядковый номер, чтобы проанализировать интенсивность мочеполовой шистосомоз инфекции у детей школьного возраста в провинции Лусака, Замбии

Christopher Simoonga, Lawrence N. Kazembe

РЕФЕРАТ

Фон: Мочеполовой шистосомоз является серьезной проблемой общественного здравоохранения в Замбии на протяжении многих лет. Однако, профилем заболевания могут различаться в разных локали из-за изменения экосистем, что способствует риску заражения

болезнью. Цель данного исследования заключалась в количественной оценке факторов риска, связанных с интенсивностью мочеполового шистосомоза инфекции у детей школьного возраста в провинции Лусака, Замбии, чтобы лучше понять местные передачи.
Методы: Данные были получены от 1 912 школьников, в 20 населенных пунктах, в районах Луангва и Кафуэ в провинции Лусака. Как индивидуальные, так и на уровне общин ковариаты были включены в порядковой логистической регрессионной модели для прогнозирования вероятности инфекции определенной интенсивности в три категории решений ответ: 0=Нет инфекции, 1=светло-инфекцией, а 2=умеренный/тяжелый зараза. Случайные эффекты были введены, чтобы захватить ненаблюдаемой гетерогенности.
Результаты: В целом, риск развития мочеполового шистосомоза была сильно связана с возрастом, высоте, на которой ребенок живет, и секс. Слабые взаимосвязи наблюдались с нормализованным индексом различий растительного покрова, максимальной температурой, и обилием улиток. Детальный анализ показал, что связь между интенсивностью заражения и возрастой и высотой являются конкретной категорией. В частности, интенсивность заражения была ниже у детей в возрасте от пяти до девяти лет по сравнению с теми, в возрасте от 10 до 15 лет (*OR* = 0.72, 95%*CI* = 0.51–0.99). Однако, возрастной риск изменен на разных уровнях инфекции, такие, что при сравнении детей с легкими инфекциями для тех, кто не был заражен, возраст был связан с более низким коэффициентом (category 1 vs category 0: *OR*=0.71, 95%*CI*: 0.50–0.99), но такое отношение было не значительным, если учитывать детей, которые были умеренно или сильно зараженных по сравнению с теми, с легким или нет инфекции (Категория 2 против 0 Категория: *OR*=0.96, 95%*CI*: 0.45–1.64). В целом, мы отметили, что дети, живущие в долине стали реже приобретать мочеполовой шистосомоз по сравнению с теми, кто живет в районах плато (*OR*=0.48, 95%*CI*: 0.16–0.71). Однако, Категория-специфические эффекты показали значимой связи 1 категории (легкая инфекция), тогда как в категории 2 (умеренный/высокий заражения), риск по-прежнему был значительно ниже для тех, кто живет в долине по сравнению с теми, кто живет в тех районах плато (*OR*=0.18, 95%*CI*: 0.04–0.75).
Заключение: Это исследование демонстрирует важность понимания динамики и неоднородности инфекции в деятельности Управления, а также предполагает, что, помимо хорошо изученных факторов интенсивности *Schistosoma*, различные другие факторы влияют на передачи. Программы контроля необходимо учитывать различную степень инфекционного заболевания, так что эффективные мероприятия могут быть разработаны.

Translated from English version into Russian by Hao-Qi Zhang

Utilización del modelo jerárquico de regresión ordinal para analizar la intensidad de la infección de esquistosomiasis urinaria en niños escolares en la provincia de Lusaka, Zambia.

Christopher Simoonga, Lawrence N. Kazembe

RESUMEN

Antecedentes: La esquistosomiasis urinaria ha supuesto un problema de salud pública importante en Zambia durante muchos años. Sin embargo, el perfil de la enfermedad puede variar según el

lugar debido a que el riesgo de adquirir la enfermedad depende de diferentes ecosistemas. El objetivo de este estudio fue cuantificar los factores de riesgo asociados con la intensidad de la infección de esquistosomiasis urinaria en niños escolares en la provincia de Lusaka, Zambia, para poder entender mejor el proceso de transmisión local.

Métodos: Se obtuvieron datos procedentes de 1912 niños en edad escolar, de 20 comunidades, en los distritos de Luangwa y Kafue en la provincia de Lusaka. A un modelo de regresión logística ordinal se incorporaron covariables tanto a nivel individual como comunitario para predecir la probabilidad de que una infección fuera de cierta intensidad en una respuesta de tres posibles resultados: 0=sin infección, 1=infección leve, y 2=infección moderada/grave. Se añadieron efectos aleatorios para captar la heterogeneidad no observada.

Resultados: En líneas generales, se observó que el riesgo de esquistosomiasis urinaria estaba fuertemente relacionado con la edad, la altitud en la que el niño vivía y el sexo. La relación fue débil con el índice de vegetación diferencial normalizado, la temperatura máxima y la abundancia de caracoles. Análisis más detallados mostraron que la relación entre las intensidades de infección con la edad y la altitud eran específicas para las categorías. En concreto, la intensidad de la infección fue menor en los niños con edades entre los cinco y los nueve años que entre los diez y los quince años (*OR* = 0,72, *IC* 95% = 0,51–0,99). Sin embargo, el riesgo a edades específicas cambiaba según los diferentes niveles de infección, por ejemplo al comparar niños con una infección leve con niños no infectados la edad estaba relacionaba con posibilidades más bajas (categoría 1 frente a la categoría 0: *OR*=0,71, *CI* 95%: 0,50–0,99), aunque tal relación no era significativa cuando se consideraban niños con infección moderada o grave en comparación con aquellos con infección leve o sin infección (categoría 2 frente a categoría 0: *OR*=0,96, *IC* 95%: 0,45–1,64). En general, se observó que los niños que vivían en el valle tenían menos probabilidad de adquirir esquistosomiasis urinaria que aquellos que vivían en las áreas de la meseta (*OR*=0.48, *CI* 95%: 0,16–0,71). Sin embargo, los efectos específicos de las categorías no mostraron una relación significativa en la categoría 1 (infección leve), mientras que en la categoría 2 (infecciones moderadas/graves) el riesgo seguía siendo significativamente más bajo en aquellos que vivían en el valle en comparación con los que vivían en áreas de la meseta (*OR*=0,18, *CI* 95%: 0,04–0,75).

Conclusión: Este estudio pone de manifiesto la importancia de conocer la dinámica y la heterogeneidad de la infección para los esfuerzos de control, y sugiere también que, independientemente de los factores ampliamente investigados que determinan la intensidad del *Schistosoma*, existen otros factores que también afectan a la transmisión. Para poder diseñar medidas eficaces, es necesario que los programas de control consideren las diferentes intensidades de la infección de la enfermedad.

Translated from English version into Spanish by Barbara Gutierrez Teira, through

TRANSLATORS
WITHOUT BORDERS
